# Magnocellular-dorsal pathway and sub-lexical route in developmental dyslexia

**DOI:** 10.3389/fnhum.2014.00460

**Published:** 2014-06-24

**Authors:** Simone Gori, Paolo Cecchini, Anna Bigoni, Massimo Molteni, Andrea Facoetti

**Affiliations:** ^1^Developmental and Cognitive Neuroscience Laboratory, Dipartimento di Psicologia Generale, Università degli Studi di PadovaPadova, Italy; ^2^Developmental Neuropsychology Unit, Istituto Scientifico “E. Medea” di Bosisio PariniLecco, Italy; ^3^Ophthalmological Unit, Istituto Scientifico “E. Medea” di San Vito al TagliamentoPordenone, Italy

**Keywords:** transient system, reading acquisition, phonological decoding, reading disability, visual disorder, dorsal stream

## Abstract

Although developmental dyslexia (DD) is frequently associate with a phonological deficit, the underlying neurobiological cause remains undetermined. Recently, a new model, called “temporal sampling framework” (TSF), provided an innovative prospect in the DD study. TSF suggests that deficits in syllabic perception at a specific temporal frequencies are the critical basis for the poor reading performance in DD. This approach was presented as a possible neurobiological substrate of the phonological deficit of DD but the TSF can also easily be applied to the visual modality deficits. The deficit in the magnocellular-dorsal (M-D) pathway - often found in individuals with DD - fits well with a temporal oscillatory deficit specifically related to this visual pathway. This study investigated the visual M-D and parvocellular-ventral (P-V) pathways in dyslexic and in chronological age and IQ-matched normally reading children by measuring temporal (frequency doubling illusion) and static stimuli sensitivity, respectively. A specific deficit in M-D temporal oscillation was found. Importantly, the M-D deficit was selectively shown in poor phonological decoders. M-D deficit appears to be frequent because 75% of poor pseudo-word readers were at least 1 SD below the mean of the controls. Finally, a replication study by using a new group of poor phonological decoders and reading level controls suggested a crucial role of M-D deficit in DD. These results showed that a M-D deficit might impair the sub-lexical mechanisms that are critical for reading development. The possible link between these findings and TSF is discussed.

## INTRODUCTION

Developmental dyslexia (DD) is often defined as a deficit in reading acquisition despite normal intelligence and access to conventional instruction (American Psychiatric Association [APA], 1994). According to the dual-route model (see Perry et al., 2007 for a review), written words can be processed either by the sub-lexical route or by the lexical route. The sub-lexical route is based on grapheme-to-phoneme correspondences and allows reading of unfamiliar words and pseudo-words. The lexical route is based on lexical unit correspondences and is crucial to read familiar and irregular words only. Both acquired and developmental disorders of reading have been generally discussed within this psycholinguistic framework (e.g., Castles and Coltheart, 1993). Phonological dyslexics show great difficulty in reading unfamiliar words and pseudo-words compared to known words, and this is thought to arise from damage to the sub-lexical route. In contrast, surface dyslexia is characterized by impaired reading of irregular words, and this is thought to arise from a damaged lexical route (e.g., Castles and Coltheart, 1993), potentially linked to an under-stimulation of the visual word recognition system resulting from low experience with literacy.

However, in shallow orthographies such as Italian, spelling-sound irregularity is limited to the supra-segmental level (i.e., to stress assignment). Thus, in Italian dyslexic children the increased weight of sub-lexical processing does not permit precise measurement of the efficiency of the lexical-route (see also Ruffino et al., 2014). It is crucial to note that –- regardless of spelling-sound regularity - for a beginner reader all words are at first pseudo-words because the lexical-orthographic representations still have to be developed. Accordingly, most longitudinal studies have shown that beginner readers primarily use the sub-lexical route (see Sprenger-Charolles et al., 2003 for a review).

Phonological decoding, which is typically measured by examining children’s pseudo-word reading performance, is one of the most critical skills for successful reading acquisition (e.g., Share, 1995). Interestingly, Ziegler et al. (2003) showed that dyslexics with regular (German-speaking children) and irregular (English-speaking children) spelling-to-sound correspondences present an extremely slow and serial phonological decoding mechanism. Thus, in learning to read it is of utmost importance to acquire an accurate and fluent use of the sub-lexical route (e.g., Goswami, 2000; see Vellutino et al., 2004 for a review).

Although there are a number of theories attempting to account for DD, two main views received major support. The first approach proposes that DD arises from deficits in systems that are specifically linguistic in nature. In particular, the phonological deficit theory suggests that DD arises from deficits in phonological processing (e.g., Snowling, 2000). In contrast, many authors suggest that deficits in underlying non-linguistic sensory mechanisms are the real core abnormality in DD (e.g., Stein and Walsh, 1997; Vidyasagar and Pammer, 2010 for visual deficits; Wright et al., 2000; Tallal, 2004 for auditory deficits). This theory, known as the temporal processing hypothesis is the multi-sensory (i.e., visual and auditory) version of the magnocellular dorsal (M-D) theory of DD, suggesting that children with DD have specific deficits in processing rapidly presented or brief sensory stimuli in either the visual or auditory modalities (see Farmer and Klein, 1995; Hari and Renvall, 2001 for reviews). Chiefly, the M-D temporal hypothesis explicitly claims that phonological decoding deficits in dyslexics could arise from impairments in sensory processing of visual and auditory dynamic-stimuli (e.g., Facoetti et al., 2010a,b). The well known M-D theory of DD is often referred specifically to the visual modality, and it is a comprehensive, albeit controversial account (e.g., Amitay et al., 2002; Sperling et al., 2005; Olulade et al., 2013). This theory stems from the observation that some reading disabled children are impaired in the specific visual M-D pathway (see Stein and Walsh, 1997; Boden and Giaschi, 2007; Vidyasagar and Pammer, 2010 for reviews). The M-D pathway originates in the ganglion cells of the retina, passes through the M-layer of the lateral geniculate nucleus (LGN), and finally reaches the occipital and parietal cortices (Maunsell and Newsome, 1987). The M-D stream is considered blind to colors, and responds optimally to contrast differences, low spatial frequencies, and to high temporal frequencies and motion (Livingstone and Hubel, 1987). The M-D stream seems to be impaired in individuals with DD, whereas the other major parallel pathway of the visual system, the parvocellular-ventral (P-V) stream, is intact (see Stein and Walsh, 1997; Boden and Giaschi, 2007; Vidyasagar and Pammer, 2010 for reviews). The P-V pathway is characterized by both lower temporal resolution and superior sensitivity to high spatial frequencies, and it is also sensitive to color changes (Livingstone and Hubel, 1987). Several studies showed the specificity of the M-D pathway deficit in individuals with DD in comparison to P-V processing in the normal range, suggesting the crucial role of the M-D pathway as the dominant visual stream for text reading (e.g., Lovegrove et al., 1986; Chouake et al., 2012). Dyslexics are less sensitive than typically reading controls to luminance patterns and motion displays with high temporal and low spatial frequencies (e.g., Eden et al., 1996), visual features that are primarily associated with the M-D pathway, but they perform normally on tasks primarily associated with the P-V pathway, such as those involving color and form (Merigan and Maunsell, 1993).

Most of the evidence for the visual M-D deficit theory has derived from studies of coherent dot motion perception (see Stein, 2001 for a review), which taps the cortical portion of the M-D pathway.

However, the coherent dot motion deficit is rarely found in all individuals in a dyslexic sample (e.g., Talcott et al., 2013). Children or adult poor readers may be specifically impaired in motion perception only in the presence of high external noise, but not in the presence of low external noise or when the signal is clearly defined (Sperling et al., 2006), weakening the strongest evidence for the more dorsal portion of the M-D pathway deficit in DD. In addition, pure M-D deficits have rarely been documented in dyslexic subjects (e.g., Amitay et al., 2002; Sperling et al., 2005). Disabled readers show impaired performance in non-M-D tasks requiring fine frequency discrimination, and the stimuli used in those tasks were neither modulated in time nor briefly presented (e.g., Amitay et al., 2002). Dyslexic children had difficulties detecting both gratings with high temporal frequency and low spatial frequency (i.e., M-D stimuli) and gratings with low temporal frequency and high spatial frequency (i.e., P-V stimuli) when the grating were embedded in external noise (Sperling et al., 2005). Nevertheless, these results did not falsify the evidence obtained from a large population of studies demonstrating significant and replicable differences between dyslexic and control groups and longitudinal studies in the coherent motion perception task (e.g., Cornelissen et al., 1995; Talcott et al., 2000, 2002, 2013; Boets et al., 2011). In addition, it has been reported that up to 75% of dyslexic individuals show visual temporal processing deficits (Lovegrove et al., 1986). Important literature supports the transient subsystem deficit hypothesis in DD which suggests a dissociation in sensitivity between low spatial, high temporal versus high spatial, low temporal grating stimuli (e.g., Martin and Lovegrove, 1987; Keen and Lovegrove, 2000). These grating stimuli tap into the receptive field characteristics of the M system at a retino-cortical level providing the most relevant support for the lower portion of the M-D theory deficit. Moreover, a post mortem study, in a small sample, showed that in the brain of individuals with DD the M neurons of the LGN were noticeably smaller than those found in normal readers’ brains, while the P neurons did not differ (Livingstone et al., 1991).

It should be noted that the M-D pathway terminates mainly in the posterior parietal cortex (Mishkin and Ungerleider, 1982; Merigan and Maunsell, 1993), which is the cortical region controlling selective attention in humans (Facoetti and Molteni, 2000; see Corbetta and Shulman, 2002, 2011 for reviews). Thus, a weakened or abnormal M-D input to the dorsal-stream could result in a spatial and temporal attention deficit in dyslexic children and adults (e.g., Brannan and Williams, 1987; Williams et al., 1987; Valdois et al., 1995; Cestnick and Coltheart, 1999; Hari et al., 1999, 2001; Vidyasagar and Pammer, 1999; Facoetti et al., 2000, 2001, 2005, 2006, 2008, 2010a,b; Iles et al., 2000; Buchholz and McKone, 2004; Bosse et al., 2007; Buchholz and Aimola Davies, 2007; Roach and Hogben, 2007; Ruffino et al., 2010; see Vidyasagar, 1999; Hari and Renvall, 2001; Boden and Giaschi, 2007; Vidyasagar and Pammer, 2010; Gori and Facoetti, 2014 for reviews) and specifically in dyslexics, a more severe poor pseudo-word reading ability in comparison to word reading skills (Buchholz and McKone, 2004; Facoetti et al., 2006, 2010a; Roach and Hogben, 2007; Jones et al., 2008; Ruffino et al., 2010, 2014). Children with autism spectrum disorder have shown attentional zooming-out and dorsal pathway disorders (e.g., Ronconi et al., 2012, 2013 a,b, 2014), the visual attentional deficit is now recognized as a core feature of DD (Franceschini et al., 2012, 2013; Zorzi et al., 2012; see Gori and Facoetti, 2014 for a review). The sub-lexical route is crucial for reading pseudo-words or new words during reading acquisition in all alphabetic languages, and it specifically requires serial attentional graphemic parsing (Facoetti et al., 2010a).

Recently, a new model, labeled “temporal sampling framework” (TSF), was proposed by Goswami (2011) providing a new, different and, intriguing prospect in the DD study. TSF integrates the data on the auditory processing deficit with the findings on neural oscillatory mechanisms related to the temporal sampling of speech. In short, the innovative proposal by Goswami (2011) suggests that deficits in syllabic perception at relatively low temporal frequencies (inside of the range of the delta/theta, i.e., <10 Hz) are the critical basis for the reading disability in DD (Power et al., 2013). This hypothesis is supported by the findings that show the role of neuronal oscillations in speech perception (Luo and Poeppel, 2007). Although this approach was presented as a possible neurophysiological substrate of the phonological deficit of DD the TSF it is not only limited to that but also, can easily be applied to all the stages of processing within the visual system (Vidyasagar, 2013). TSF also has the potential to integrate several low level deficits already associated with DD (Vidyasagar, 2013; Pammer, 2014). As suggested by Vidyasagar (2013) the D-stream deficit could also be integrated in the TSF theory because the TSF fits well in describing the attentional feedback within the D-stream. What is expected is a deficit in neural oscillation at higher temporal frequency than in the auditory modality because the M-D visual stream process relatively high temporal frequency (Vidyasagar, 2013).

Assuming that the M-D pathway deficit is the neurobiological basis of visual selective attention disorders in DD, we predict that the M-D deficit should be found mainly in poor phonological decoders. Therefore, the aim of the present study was to investigate the efficiency of the visual M-D pathway inside the TSF approach in dyslexic and typically reading children (age- and reading-matched) using the frequency doubling (FD) illusion. The FD illusion is a visual illusion that was first described by Kelly (1966). Measuring a visual illusion, even if it sounds counterintuitive, can be done in a very accurate way (e.g., Gori et al., 2006, 2008, 2011; Yazdanbakhsh and Gori, 2008; Giora and Gori, 2010; Gori and Spillmann, 2010; Ito, 2012). The FD illusion appears to be dependent on the spatial and temporal frequency of a flickering grating. When a grating with a spatial frequency of 0.1–4 c/deg is flickering faster than 15 Hz, the viewer perceives a grating with double the physical spatial frequency. The FD was later explained by Kelly (1981) in terms of the full wave rectification carried out by the visual system. Such rectification is found in M(y)-cells of the primate retina (Benardete et al., 1992) and LGN (Kaplan and Shapley, 1982; Marrocco et al., 1982). It is therefore suggested, that responses from the M(y)-cells underlie perception of the FD illusion (see Maddess et al., 1992 for a detailed discussion regarding the relationship between M(y)-cells and frequency doubling). A previous study (Pammer and Wheatley, 2001) showed that individuals with DD are less sensitive to the FD illusion than normal readers, supporting a low-level deficit in the M-D pathway. Kevan and Pammer (2008) demonstrated that children at risk of DD already show a higher threshold for the FD illusion even at the pre-reading stage. Importantly, the threshold for the FD illusion at the pre-reading stage predicts future reading skills (Kevan and Pammer, 2009). The FD illusion is, therefore, a consolidated M-D index which taps the lower portion of the M-D pathway and can be difficultly described in terms of signal-to-noise exclusion (e.g., Sperling et al., 2005, 2006; Olulade et al., 2013). Interestingly, the FD illusion was never previously tested in children with DD in shallow languages as Italian. More importantly, the FD illusion is a temporal stimulus that fit well with the opportunity to measure the M-D pathway functionality inside the context of the TSF. What is expected, indeed, is that if a neural oscillation deficit is present also in the visual system of children with DD and specifically in their M-D stream (Vidyasagar, 2013), the children with DD will need more contrast to perceive the flickering stimulus at the same oscillation frequency in comparison with the chronological-age control group.

In addition, we studied the efficiency of the P-V pathway in the same children to rule out the alternative explanation that perceptual processing is generally inefficient in dyslexic children (e.g., because of poor perceptual noise exclusion). The task employed was High-Pass Resolution (HPR) perimetry which measures the detection threshold for fixed ring-shaped stimuli of different sizes. HPR perimetry is commonly adopted for selective analysis of the lower portion of the P-V pathway.

A crucial aim of this study was to investigate if a specific subgroup of children with DD, i.e., poor phonological decoders, were affected by the M-D deficit. In the Experiment 1 we selected a poor phonological decoder subgroup and we compared them with the chronological-age control group. In the Experiment 2 we collected a new poor phonological decoder group in order to carry out a replication study. Stringently, we contrast the new group with a reading-level (RL) control group. The RL children (see Goswami, 2003) were never included in previous studies using FD illusion. The inclusion of the RL group is particularly important to address the issue of the causal link between FD illusion perception and poor phonological decoding.

## EXPERIMENT 1

### METHODS

#### Participants

Seventeen dyslexic children (mean age 11 years, SD = 2), were selected from a sample of children referred to the Neuropsychiatric Unit of the scientific Hospital “E. Medea” of San Vito al Tagliamento, Pordenone, Italy, because of specific reading disability. These children had been diagnosed as dyslexic based on standard criteria (American Psychiatric Association [APA], 1994). Their performance reading aloud had to be two standard deviations below the norm in one reading subtest or 1 standard deviation below the norm in at least two reading subtests according to the Italian age-standardized tests (Sartori et al., 1995). The ability to read aloud was measured using a clinical standardized Italian test composed of 112 words (separated into four lists; word reading task, Sartori et al., 1995) and phonological decoding ability was measured using three standardized clinical lists of 48 Italian pseudo-words (pseudo-word Reading task, Sartori et al., 1995). Finally, reading fluency and errors in age-standardized prose passages from Italian clinical tests were used to measure ecological-context reading (Sartori et al., 1995).

The children with DD were selected on the basis of:

(1) a full-scale IQ greater than, or equal to, 85, as measured by the Wechsler intelligence scale for children-revised (WISC-R; Wechsler, 1986);
(2) normal or corrected-to-normal vision and hearing;
(3) absence of neurological and/or psychiatric disorders;
(4) absence of specific language impairments (American Psychiatric Association [APA], 1994); and
(5) absence of attention deficit disorders with or without hyperactivity (ADHD and ADD; American Psychiatric Association [APA], 1994). Several recent studies have shown that sustained attention deficits are significant covariates in group studies using dyslexics and controls pointing out the relevance of this exclusion criterion (e.g., Rochelle et al., 2009).

The children with DD were divided into two subgroups (i.e., poor and non-impaired phonological decoders) according to their performance on the pseudo-word reading test (Sartori et al., 1995). Children were considered poor phonological decoders (PPD, *n* = 12 dyslexic children) if their performance, in terms of mean between accuracy and speed of pseudo-word reading, was below two standard deviation from the norm. The remaining children were assigned to the non-impaired phonological decoders subgroup (NPD, *n* = 5 dyslexic children). Note that pseudo-word reading efficiency is the most appropriate measure of phonological decoding skills. A performance well below the normative data implies that the child is a poor phonological decoder (e.g., Facoetti et al., 2006, 2010a; Ruffino et al., 2010, 2014). In dyslexic children with a regular spelling-to-sound correspondence, like Italian, it is practically impossible to apply the classical English sub-typing (i.e., phonological and surface DD; e.g., Castles and Coltheart, 1993; Talcott et al., 2013) because the English language presents an higher number of irregular words. Importantly, the two dyslexic groups did not differ in word reading [*t*_(14)_ = 0.68, *p *> 0.05] nor in text reading [*t*_(14)_ = -0.01, *p *> 0.05] abilities, excluding that our PPDs were simply more severely impaired dyslexic children.

Twenty four chronological, age- and IQ-matched typically reading children (mean age 10 years, SD = 2) were randomly selected from the same primary school. They were of average or above average intelligence on three WISC-R (Wechsler, 1986) sub-tests (i.e., Block Design and Comprehension).

All participants’ parents gave informed consent.

**Table 1** shows the mean and SD of age, Block Design, Comprehension and text reading tests for the control and dyslexic groups. Controls and dyslexics were comparable to chronological age and IQ. In contrast, controls and dyslexics were significantly different on accuracy and speed of word and text reading. In **Table 2** we showed that PPD and NPD groups differed only for the pseudo-word reading mean (accuracy and speed).

**Table 1:** Mean (M) and standard deviation (SD) of age, Comprehension, Block Design sub-test (WISC-R; Wechsler, 1986), text reading errors and speed in the control and dyslexic groups.

	Controls (*N* = 24)		Dyslexics (*N* = 17)		Comparison	
	*M*	SD	*M*	SD	*T* (39)	*P*
Age (months)	124	21	128	27	-0.46	0.65
Comprehension (standard score)	10.9	2.3	12	2.6	-1.49	0.14
Block design (standard score)	13.8	2.2	12.5	2.9	1.71	0.09
Text reading errors (number) (*Z*-score)	2.1, 0.54	2.5, 0.56	10.7, -1.05	6.7, 1.19	-5.78, -5.66	<0.001, <0.001
Text reading speed (s) (*Z*-score)	33, 0.31	20.7, 0.48	82, -2.58	49.9, 1.5	-4.31, -8.81	<0.001, <0.001

**Table 2:** Mean (M) and standard deviation (SD) of Comprehension, Block Design sub-test (WISC-R; Wechsler, 1986), text reading mean (errors and speed), word reading mean (errors and speed), pseudo-word reading mean (errors and speed) in the two dyslexic subgroups: the poor phonological decoders (PPD) and the non-impaired phonological decoders (NPD) groups.

	PPD (*N* = 12)		NPD (*N* = 5)		Comparison	
	*M*	SD	*M*	SD	*T* (15)	*P*
Comprehension (standard score)	12.5	2.58	11	2.45	1.11	0.28
Block design (standard score)	12.67	2.64	12	2.67	0.42	0.68
Text reading mean (*Z*-score)	-1.66	0.85	-2.15	1.02	-1.02	0.33
Word reading mean (*Z*-score)	-4.03	1.95	-3.33	1.86	0.68	0.51
Pseudoword reading mean (*Z*-score)	-3.26	1.09	-0.92	0.67	4.39	<0.001

All participants underwent a complete ophthalmological evaluation, consisting of “Early Treatment Diabetic Retinopathy Study” (ETDRS) chart (standardized eye charts and visual acuity test), orthoptic examination, anterior segment slit lamp examination, cycloplegic refraction, and indirect ophthalmoscopy.

#### Apparatus and stimuli

***Frequency doubling perimetry.*** The FD perimetry relies on the frequency doubling illusion described in the Introduction. The settings resembled the ones adopted, in a previous study, by Pammer and Wheatley (2001). A low spatial frequency grating displayed in counter-phase flicker mode at a high temporal frequency is perceived as if it had twice its actual spatial frequency. The Humphrey Matrix perimeter was the presentation tool used with the program threshold set to 30-2. The threshold is expressed as contrast attenuation in decibels (dB) and it is calculated by a staircase algorithm built into the Humphrey Matrix perimeter tool. Thresholds ranged from 0 to 38 db. The stimulus was presented at 69 locations in the 30 central degrees of the visual field. The background luminance was 100 cd/m^2^. The pattern consisted of a sinusoidal grating presented at different contrast levels, arranged in 5° × 5° square stimuli, and a circular macular stimulus of 2.5° radius. The spatial frequency of the bar target was 0.50 cy/deg, the counter-phase flickered at 18 Hz and was presented for 300 ms. The FD provides selective stimulation of the M-retinal ganglion cells and M LGN neurons. Due to the design of the target, no P-cell activity should be stimulated. Visual fields with 20% or less false positive or false negative responses, and 30% or less fixation errors were considered acceptable.

***High-pass resolution (ophthimus) perimetry.*** The Ophthimus system HPR perimetry uses ring-shaped stimuli, consisting of dark borders and a lighter core. Fourteen different sized targets are available (ranged from 1.26 to 17.64 dB). The target contrast was held constant while the size varied in steps of 1.26 dB. The background luminance was 20 cd/m^2^. The luminance of the ring borders was 15 cd/m^2^ and the luminance of the ring core was 25 cd/m^2^. The target was “high-pass spatial frequency filtered”. The participants either detected and resolved it, or it was invisible to them. The perimeter assesses resolution thresholds as the smallest stimulus size seen in the 50 locations over the central 30° of the visual field. The blindspot is not mapped. The high-pass spatial frequency filter allows for selective analysis of the P-cells of the retina and probably of the LGN. Due to the software characteristics, fixation errors were not tested for the HPR perimetry.

#### Procedures

All participants performed the FD and the HPR perimetry in random order, beginning with one of the two eyes. On another day, they performed the two visual field tests again, beginning with the other eye, in order to avoid fatigue and any learning effects. The children were verbally instructed on how to perform the two tests and were given the opportunity to practice. Two pauses were given throughout each test, and a 5-min pause was permitted between testing of the first and second eye.

Each child was seated comfortably with their face against the eyepiece. For the FD testing the child was given a description of the display, and instructed to press the response button each time she/he saw a pattern against the homogeneous background. For the HPR task each child had to report whenever they saw a circle in any tested position of the visual field.

### RESULTS

#### FD results

**FIGURE 1:**
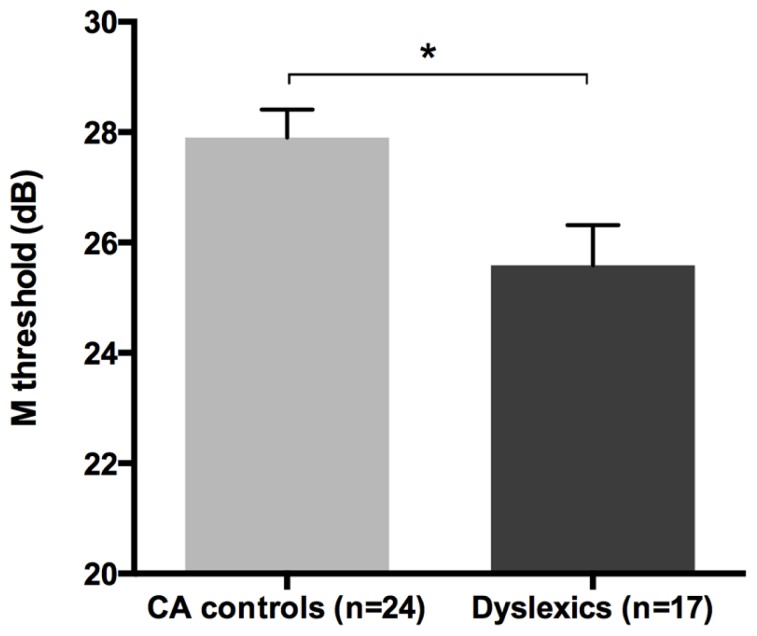
**The difference between the chronological age- and IQ-matched (CA) controls and dyslexic children in the FD task measuring the M-D pathway functionality (i.e., M threshold) in the Experiment 1.** ^*^p < 0.05.

**FIGURE 2:**
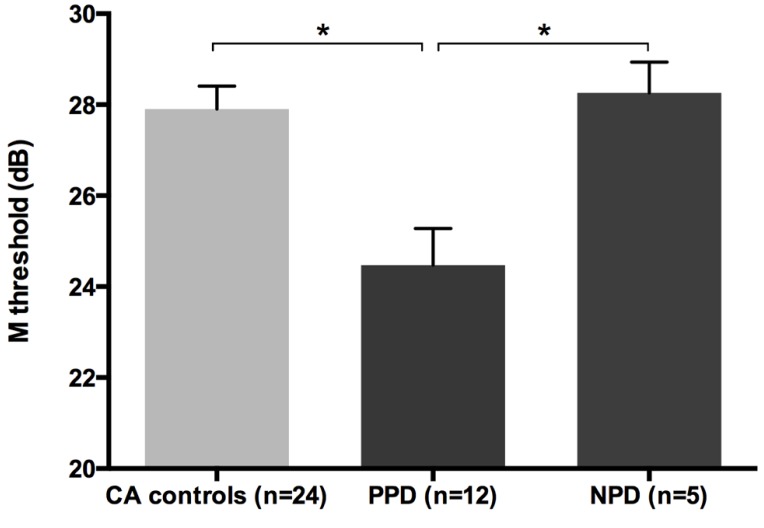
**The difference between chronological-age and IQ matched (CA) controls and the subtypes of children with DD (i.e., poor phonological decoders, hereafter PPD and non-impaired phonological decoders, hereafter NPD) in the FD task (Experiment 1).** ^*^p < 0.05.

All groups and subgroups were normally distributed as showed by a non-significant Shapiro-Wilk test of normality (all *p*s > .05). The mean FD thresholds (averaged for all positions and the two eyes) for the children with DD (*n* = 17) differed significantly [*t*-test: *t*_(39)_ = 2.697, *p* < 0.05] in comparison to the normal reader age- and IQ-matched controls (*n* = 24), showing that the dyslexic group was less sensitive to the FD illusion at 18 Hz of temporal frequency of (see the **Figure 1** and the plot of the individual data in **Figure 6A**). An univariate ANCOVA (omnibus test) was run in which the independent variable was group (chronological age- and IQ-matched controls, PPDs and NPDs) and the dependent variable was the mean FD threshold, co-varied for the participant’s age [*F*_(3,37) _= 11.999, *p* < 0.05, $\eta_{p}^{2}$ = 0.493; see **Figure 2** and the plot of the individual data in **Figure 6A**]. A planned comparison (univariate ANCOVA) was then run where the independent variable was group (chronological age- and IQ-matched controls vs. PPDs) and the dependent variable was the mean FD threshold, co-varied for the participants’ ages [*F*_(2,33)_ = 15.139, *p* < 0.05, $\eta_{p}^{2}$ = 0.478]. Finally another planned comparison (univariate ANCOVA), where the independent variable was the group (age- and IQ-matched controls vs. NPDs) and the dependent variable was the mean FD threshold, co-varied for the participants’ ages [*F*_(1,26) _= 1.899, *p* > 0.05, $\eta_{p}^{2}$ = 0.068]. In summary, only the PPDs were significantly worse than the controls in their mean FD threshold.

**FIGURE 3:**
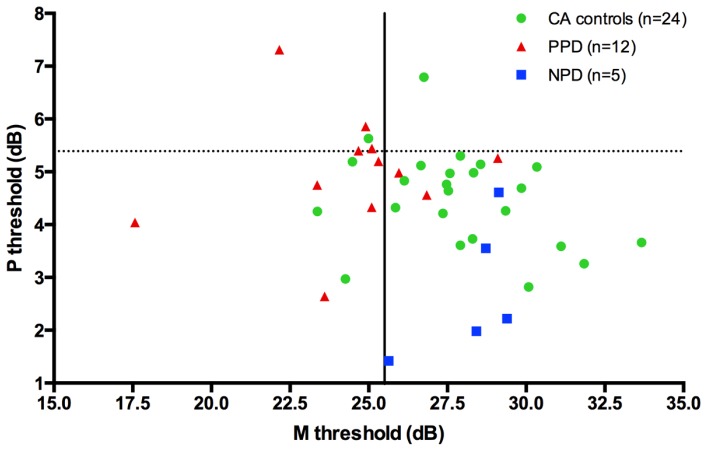
**The scatter-plot shows that at individual level the 75% of the PPDs (the red triangle) are at least 1 SD under the mean of the CA controls (the green circle) in the FD task (Experiment 1)**.

Although PPD children showed a significantly worse performance in the FD task at the group level, in comparison with the controls, it is important to establish how reliable is this abnormal pattern at the individual level. In the PPD group, 75% of them were at least 1 SD below the mean of the controls (**Figure 3**).

Based on these results indicating a specific relationship between M-D pathway and the spelling to sound translation process we concentrate our Experiment 2 only on the PPDs.

#### HPR results

**FIGURE 4:**
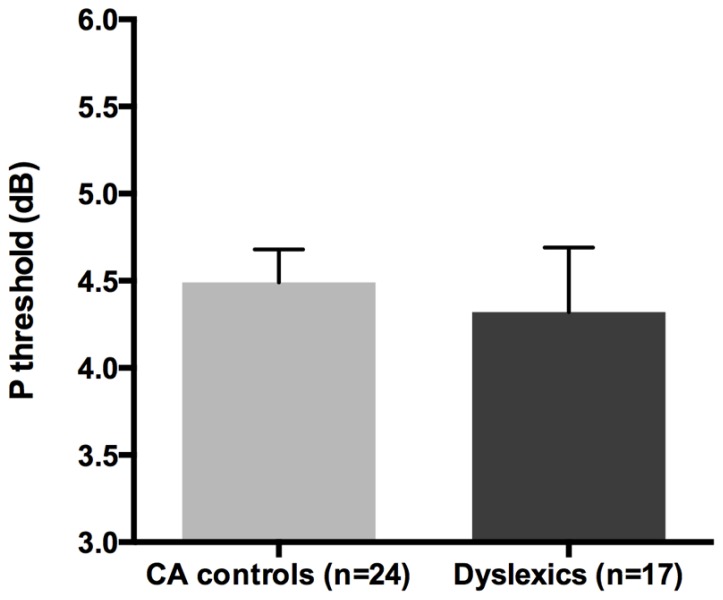
**No difference was found between age and IQ matched (CA) controls and the children with DD in the HPR task measuring the P-V pathway functionality (i.e., P threshold) in the Experiment 1**.

All groups and subgroups were normally distributed as showed by a non-significant Shapiro-Wilk test of normality (all *p*s > 0.05). The mean HPR thresholds (averaged for all positions and the two eyes) for the children with DD (*n* = 17) and the normal reader age- and IQ-matched controls (*n* = 24) did not differ significantly [*t*-test: *t*_(39)_ = 0.432, *p* > 0.05] showing that the dyslexic and normal reader groups were similar in their P-V pathway performance (see **Figure 4** and the plot of the individual data in **Figure 6B**).

#### The relationship between M-D functioning and reading text ability

Partial correlation between M-D pathway functioning (indexed by FD threshold) and the text reading efficiency (the mean between speed and errors z-scores) in the entire sample (*n* = 40; a child with DD score is missing), controlling for chronological age, IQ (block design and similarities), and the P-V functioning, was significant (*r* = 0.43, *p* < 0.01).

To determine the predictive relationships between M-D pathway functioning and ecological reading skills in a more stringent way, we computed a three-step fixed-entry multiple regression analysis in which the dependent variable was text reading efficiency. To control for the effects of chronological age, verbal and nonverbal IQ, and P-V pathway functioning, the predictors entered at the three steps were as follows: (1) age, block design and similarities, (2) P-V pathway threshold, and (3) M-D pathway threshold. The ANOVA regression model was significant [*F*_(5,34)_ = 2805, *p* < 0.05] explaining the 29% of the text reading quote of variance. Only the M-D pathway measure, entered last, accounted for a significant quote of unique variance in text reading efficiency (*r*^2^ change = 0.16, *p* < 0.01).

## EXPERIMENT 2

### METHODS

#### Participants

In a replication study, we selected a new sample of 8 PPDs (mean age 11 years, SD = 2) and a RL matched control group (10 younger children well matched to the dyslexics for reading ability and IQ, mean age 7 years, SD = 1). In order to find an RL group in Italian speaking language population, it is necessary to search for younger children than would be use in countries with deeper orthographic-to-phonological mapping than in Italian. All the inclusion criteria were the same for the Experiment 1. For details of the new two groups check **Table 3**. All participants’ parents gave informed consent.

**Table 3:** Mean (M) and standard deviation (SD) of chronological age, Block Design sub-test (WISC-R; Wechsler, 1986), phonological decoding speed and errors in the reading-level (RL) controls and poor phonological decoders (PPD) of the Experiment 2.

	RL controls (*N* = 10)		PPD (*N* = 8)		Comparison	
	*M*	SD	*M*	SD	*T* (16)	*P*
Block design (standard score)	11.5	1.43	11	1.51	0.72	0.48
Chronological age (months)	91.5	5.4	126.2	8.67	10.41	0.001
Pseudo-word reading speed (s)	54.5	14.96	64.12	25.38	1.01	0.33
Pseudo-word reading errors (errors)	7.3	2.54	7.9	2.74	0.46	0.65

#### Apparatus and stimuli

***Frequency doubling perimetry.*** The procedure was exactly the same for the Experiment 1. The HPR perimetry task was not performed because the results were not be discriminative in the Experiment 1.

### RESULTS

**FIGURE 5:**
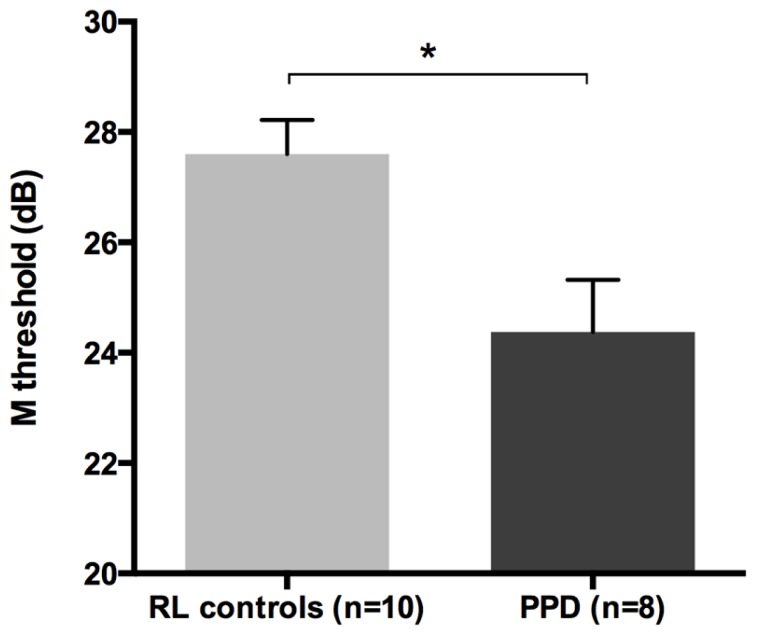
**The difference between the new sample of PPDs and reading-level and IQ-matched (RL) controls in the FD task (Experiment 2).** ^*^p < 0.05.

**FIGURE 6:**
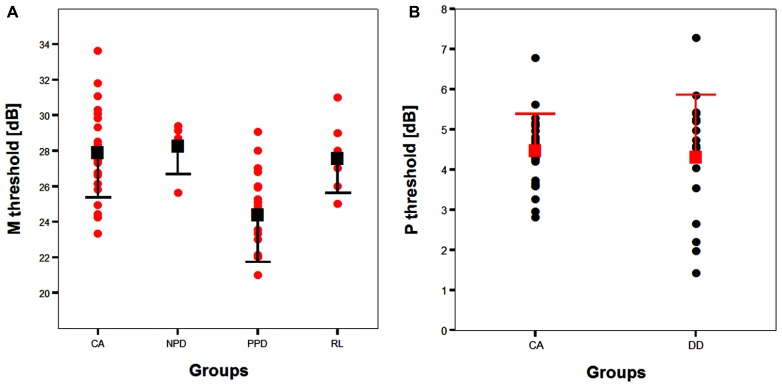
**(A)** The scatter-plot of the individual data showing the difference between the children without DD (i.e., CA and RL controls) and the subtypes of children with DD (NPD and PPD) in the FD task measuring the M-D pathway functionality (Experiment 1 and 2). The findings did not change running the ANCOVA without the outliers. Error bars represent one standard deviation of the mean. **(B)** The plot of the individual data showing the difference between the children without (i.e., CA controls) and with DD in the HPR task (Experiment 1). Error bars represent one standard deviation of the mean.

The mean FD thresholds (averaged for all positions and the two eyes) for PPDs (*n* = 8) and the typical readers IQ- and RL matched controls (*n* = 10) differed significantly [*t*-test: *t*_(16) _= 2.962, *p* < 0.05] showing that the PPDs were less sensitive to the FD illusion at 18 Hz of temporal frequency even compared to younger normal readers with the same reading abilities (see **Figure 5** and the plot of the individual data in **Figure 6A**). The two control groups (CA and RL) did not differ in the FD illusion threshold (*p* > 0.05). It is not surprising that age did not affect that task because the M-D pathway should be completely operative much before the age of 7 years (e.g., Gori et al., in press).

Although the study was designed as two independent experiments the PPDs of the Experiment 1 and 2 were very similar in age and they did not differ in other relevant variables. Additionally, we found it could be of interest to merge the two groups of PPDs and contrast them with the CA and the RL controls. We applied an univariate ANOVA with the FD threshold as the independent variable and groups (PPD, *n* = 25, CA, *n* = 24 and RL, *n* = 10) as the between subjects factor. The Group main effect was significant [*F*_(2,56)_ = 7.33; *p* < 0.05] and PPD group differed from CA and from RL (Bonferroni multiple comparison with *p*s < 0.05). This result confirmed the results obtained separately in Experiment 1 and 2.

## DISCUSSION

These results provide strong support for an M-D deficit in DD that has its origins at the sub-cortical level of the pathway (i.e., at the LGN). Notably, this deficit characterized only a subgroup of dyslexics, namely the poor phonological decoders.

The absence of differences in the P-V task between groups emphasizes the selectivity of the visual deficit that seems to be associated with the reading disability in the sub-lexical route. It is therefore impossible to attribute the difference found in the FD task to poor testing endurance, as the children with DD performed as well as the typically reading children on the HPR task. The absence of a difference between the two groups in the P-V task cannot be attributed to a ceiling or a floor effect that could mask a poor performance of children with DD as shown by the individual data shown in **Figure 6B**. The graph shows that the data of both groups were not clustered on the limits of the stimulation range. Consequently, even if the M-D and the P-V task could be different in difficulty, both tasks should have enough sensitivity to show a possible difference between groups.

Moreover, the poor phonological decoders with DD not only demonstrated less sensitivity to the FD illusion compared to the typical reading age- and IQ-matched group (supporting Pammer and Wheatley, 2001), but also to a reading-level and IQ-matched control group. The latter result excludes that the M-D deficit is simply an effect of DD.

This is the first study to show that the M-D deficit in children with DD is present also when compared with younger children with the same reading performance, challenging the idea that the M-D deficit is a simple effect of reading deprivation and not a cause, as recently suggested by Olulade et al. (2013). Importantly, the results obtained in the between-group analyses cannot be attributed to the presence of a few (and perhaps peculiar) dyslexic children in the PPD group but were fully confirmed by the analyses at the level of individual cases. M-D deficits were present in a large part of the PPDs (75%) in comparison to CA. Moreover, none of the NPDs presents a deficit in the FD task. Thus, the FD task allowed us to accurately discriminate between poor phonological decoders and CA controls. Regardless of whether children in the NPD group constitute a specific subtype in shallow orthographies (e.g., Wimmer, 1993) or they have partly compensated for their reading deficit, M-D pathway functioning seems to play an important role in phonological decoding. In general, the results from the current study provide evidence for an M-D stream involvement in DD characterized by poor phonological decoding (the most frequent pattern even in Italian individuals with DD; see Facoetti et al., 2006). This conclusion was supported by the finding that individual differences in the M-D pathway were predictive of reading performance even after controlling for age, IQ, and P-V pathway functioning. The FD task allowed us to show an M-D deficit that seems to be in the lower portion of this visual stream (the sub-cortical component and/or the primary visual cortex) and that cannot be interpreted as a signal-to-noise exclusion deficit (which could be consider the weak point of the coherent dot motion task) or as an effect of reading level (as clearly highlighted by the difference with the RL group). The present results could be interpreted inside the framework of the TSF (Goswami, 2011). A multi-sensory (auditory and visual) temporal sampling disorder of neural oscillations could include the M-D deficit theory as one of several possible causes of DD. While an auditory deficit in low temporal frequency is observed in DD (e.g., Goswami, 2011; Power et al., 2013), in the visual modality a higher temporal frequency processing seems to be damaged in children with DD which is in agreement with the Vidyasagar (2013) prediction of a temporal oscillation deficit in the M-D pathway. However, the spatial and temporal sampling of the orthographic information could also be considered as a proxy deficit for auditory modality deficits in phonological decoding (e.g., Vidyasagar and Pammer, 2010). Moreover, as recently suggested (Vidyasagar, 2013; Pammer, 2014) the TSF seems to be very appropriate to model not only for the auditory deficits of DD but also the visual deficits that appear to be common in DD. Our results, based on temporal illusion sensitivity seem to be the first experimental test of the TSF in visual modality. The fact that children with DD need more contrast to see the FD pattern at 18 hz of temporal frequency, in comparison with both control groups supports the hypothesis of a neural oscillation deficit in the M-D pathway of children with DD. This neural oscillation defict seems to be selective for the M-D stream as theorized by Vidyasagar (2013).

Further research is now necessary to better understand the role of the cortical component of the M-D pathway in DD. In order to pursue that goal a sensitivity task employing specific motion illusions (i.e., Gori and Hamburger, 2006; Gori et al., 2006, 2010b, 2011; Gori and Yazdanbakhsh, 2008; Yazdanbakhsh and Gori, 2008, 2011; Hamburger, 2012) could be devised given the fact that this kind of illusory motion is processed by V5/MT (Ruzzoli et al., 2011). Potential differences in this task could not be related to a general perceptual noise exclusion deficit.

In conclusion, sensitivity to the FD illusion could provide a simple and powerful diagnostic tool for the evaluation and identification of the risk of DD, even at the pre-reading stage (Kevan and Pammer, 2008, 2009) and the results obtained with the RL group strongly point in the direction of a causal role of a neural oscillatory deficit in the M-D pathway of individuals with DD.

## Conflict of Interest Statement

The authors declare that the research was conducted in the absence of any commercial or financial relationships that could be construed as a potential conflict of interest.

## References

[B1] American Psychiatric Association (APA). (1994). Task Force on DSM-IV. Diagnostic and Statistical Manual of Mental Disorders. Washington, DC: American Psychiatric Press 886

[B2] Amitay S. Ben-Yehudah G. Banai K. Ahissar M. (2002). Disabled readers suffer from visual and auditory impairments but not from a specific magnocellular deficit. *Brain* 125 2272–2285 10.1093/brain/awf231 12244084

[B3] Benardete E. A. Kaplan E. Knight B. W. (1992). Contrast gain control in the primate retina: P cells are not X-like, some M cells are. *Vis. Neurosci.* 8 483–486 10.1017/S0952523800004995 1586649

[B4] Boden C. Giaschi D. (2007). M-stream deficits and reading-related visual processes in developmental dyslexia. *Psychol. Bull.* 133 346–366 10.1037/0033-2909.133.2.346 17338604

[B5] Boets B. Vandermosten M. Cornelissen P. Wouters J Ghesquière P. (2011). Coherent motion sensitivity and reading development: changing relations in the transition from pre-reading to reading stage. *Child Dev.* 82 854–869 10.1111/j.1467-8624.2010.01527.x 21410913

[B6] Bosse M. Tainturier M. J. Valdois S. (2007). Developmental dyslexia: the visual attention span deficit hypothesis. *Cognition* 104 198–230 10.1016/j.cognition.2006.05.009 16859667

[B7] Brannan J. R. Williams M. C. (1987). Allocation of visual attention in good and poor readers. *Percept. Psychophys.* 41 23–28 10.3758/BF03208209 3822740

[B8] Buchholz J Aimola Davies A. (2007). Attentional blink deficits observed in dyslexia depend on task demands. *Vision Res.* 47 1292–1302 10.1016/j.visres.2006.11.028 17408718

[B9] Buchholz J. McKone E. (2004). Adults with dyslexia show deficits on spatial frequency doubling and visual attention tasks. *Dyslexia* 10 24–43 10.1002/dys.263 14998141

[B10] Castles A. Coltheart M. (1993). Varieties of developmental dyslexia. *Cognition* 47 149–180 10.1016/0010-0277(93)90003-E 8324999

[B11] Cestnick L. Coltheart M. (1999). The relationship between language-processing and visual-processing deficits in developmental dyslexia. *Cognition* 71 231–255 10.1016/S0010-0277(99)00023-2 10476605

[B12] Chouake T. Levy T. Javitt D. C. Lavidor M. (2012). Magnocellular training improves visual word recognition. *Front. Hum. Neurosci. * 10:6–14 10.3389/fnhum.2012.00014 PMC3277270 22363277

[B13] Corbetta M. Shulman G. L. (2002). Control of goal-directed and stimulus-driven attention in the brain. *Nat. Rev. Neurosci.* 3 201–215 10.1038/nrn755 11994752

[B14] Corbetta M. Shulman G. L. (2011). Spatial neglect and attention networks. *Annu. Rev. Neurosci.* 34 569–599 10.1146/annurev-neuro-061010-113731 21692662 PMC3790661

[B15] Cornelissen P. Richardson A. Mason A. Fowler S. Stein J. (1995). Contrast sensitivity and coherent motion detection measured at photopic luminance levels in dyslexics and controls. *Vision Res.* 35 1483–1494 10.1016/0042-6989(95)98728-R 7645277

[B16] Eden G. F. VanMeter J. W. Rumsey J. M. Maisog J. M. Woods R. P. Zeffiro T. A. (1996). Abnormal processing of visual motion in dyslexia revealed by functional brain imaging. *Nature* 382 66–69 10.1038/382066a0 8657305

[B17] Facoetti A. Lorusso M. L. Cattaneo C. Galli R. Molteni M. (2005). Visual and auditory attentional capture are both sluggish in children with developmental dyslexia. *Acta Neurobiol. Exp.* 65 61–72 10.55782/ane-2005-1540 15794032

[B18] Facoetti A. Molteni M. (2000). Is attentional focusing an inhibitory process at distractor location? *Brain Res. Cogn. Brain Res.* 1–2 185–188 10.1016/S0926-6410(00)00031-8 10978707

[B19] Facoetti A. Paganoni P. Turatto M. Marzola V. Mascetti G. G. (2000). Visual-spatial attention in developmental dyslexia. *Cortex* 36 109–123 10.1016/S0010-9452(08)70840-2 10728901

[B20] Facoetti A. Ruffino M. Peru A. Paganoni P. Chelazzi L. (2008). Sluggish engagement and disengagement of non-spatial attention in dyslexic children. *Cortex* 44 1221–1233 10.1016/j.cortex.2007.10.007 18761136

[B21] Facoetti A. Trussardi A. N. Ruffino M. Lorusso M. L. Cattaneo C. Galli R. (2010a). Multisensory spatial attention deficits are predictive of phonological decoding skills in developmental dyslexia. *J. Cogn. Neurosci.* 22 1011–1025 10.1162/jocn.2009.21232 19366290

[B22] Facoetti A. Corradi N. Ruffino M. Gori S. Zorzi M. (2010b). Visual spatial attention and speech segmentation are both impaired in preschoolers at familial risk for developmental dyslexia. *Dyslexia* 16 226–239 10.1002/dys.413 20680993

[B23] Facoetti A. Turatto M. Lorusso M. L. Mascetti G. G. (2001). Orienting of visual attention in dyslexia: evidence for asymmetric hemispheric control of attention. *Exp. Brain Res.* 138 46–53 10.1007/s002210100700 11374082

[B24] Facoetti A. Zorzi M. Cestnick L. Lorusso M. L. Molteni M. Paganoni P. (2006). The relationship between visuo-spatial attention and nonword reading in developmental dyslexia. *Cogn. Neuropsychol.* 23 841–855 10.1080/02643290500483090 21049356

[B25] Farmer M. E. Klein R. M. (1995). The evidence for a temporal processing deficit linked to dyslexia: a review. *Psychon. Bull. Rev.* 2 460–493 10.3758/BF03210983 24203785

[B26] Franceschini S. Gori S. Ruffino M. Pedrolli K. Facoetti A. (2012). A causal link between visual spatial attention and reading acquisition. *Curr. Biol.* 22 814–819 10.1016/j.cub.2012.03.013 22483940

[B27] Franceschini S. Gori S. Ruffino M. Viola S. Molteni M. Facoetti A. (2013). Action video games make dyslexic children read better. *Curr. Biol.* 23 462–466 10.1016/j.cub.2013.01.044 23453956

[B28] Giora E. Gori S. (2010). The perceptual expansion of a filled area depends on textural characteristics. *Vision Res.* 50 2466–2475 10.1016/j.visres.2010.08.033 20801140

[B29] Gori S. Facoetti A. (2014). Perceptual learning as a possible new approach for remediation and prevention of developmental dyslexia. *Vision Res.* 99 78–87 10.1016/j.visres.2013.11.011 24325850

[B30] Gori S. Giora E. Agostini T. (2010a). Measuring the Breathing Light Illusion by means of induced simultaneous contrast. *Perception* 39 5–12 10.1068/p6489 20301842

[B31] Gori S. Giora E. Stubbs D. A. (2010b). Perceptual compromise between apparent and veridical motion indices: the unchained-dots illusion. *Perception* 39 863–866 10.1068/p6678 20698480

[B32] Gori S. Giora E. Pedersini R. (2008). Perceptual multistability in figure-ground segregation using motion stimuli. *Acta Psychol.* 129 399–409 10.1016/j.actpsy.2008.09.004 18929348

[B33] Gori S. Giora E. Yazdanbakhsh A. Mingolla E. (2011). A new motion illusion based on competition between two kinds of motion processing units: the accordion grating. *Neural Netw.* 24 1082–1092 10.1016/j.neunet.2011.06.017 21784613

[B34] Gori S. Hamburger K. (2006). A new motion illusion: the rotating-tilted-lines illusion. *Perception* 35 853–857 10.1068/p5531 16836050

[B35] Gori S. Hamburger K. Spillmann L. (2006). Reversal of apparent rotation in the Enigma-figure with and without motion adaptation and the effect of T-junctions. *Vision Res.* 46 3267–3273 10.1016/j.visres.2006.03.009 16757011

[B36] Kaplan Gori S. Mascheretti S. Giora E. Ronconi L. Ruffino M. Quadrelli E. (in press) The DCDC2 intron 2 deletion impairs illusory motion perception unveiling the selective role of magnocellular-dorsal stream in reading (dis)ability. *Cereb. Cortex.*. 10.1093/cercor/bhu234 25270309

[B37] Gori S. Spillmann L. (2010). Detection vs. grouping thresholds for elements differing in spacing, size and luminance. An alternative approach towards the psychophysics of Gestalten. *Vision Res.* 50 1194–1202 10.1016/j.visres.2010.03.022 20363241

[B38] Gori S. Yazdanbakhsh A. (2008). The riddle of the rotating-tilted-lines illusion. *Perception* 37 631–635 10.1068/p5770 18546670

[B39] Goswami U. (2003). Why theories about developmental dyslexia require developmental designs. *Trends Cogn. Sci.* 7 534–540 10.1016/j.tics.2003.10.003 14643369

[B40] Goswami U. (2000). Phonological representations, reading development and dyslexia: towards a cross-linguistic theoretical framework. *Dyslexia* 6 133–151 10.1002/(SICI)1099-0909(200004/06)6:2<133::AID-DYS160>3.0.CO;2-A 10840513

[B41] Goswami U. (2011). A temporal sampling framework for developmental dyslexia. *Trends Cogn. Sci.* 15 3–10 10.1016/j.tics.2010.10.001 21093350

[B42] Hamburger K. (2012). Still motion? Motion illusions and luminance contrast. *Perception* 41 113–116 10.1068/p7005 22611668

[B43] Hari R. Renvall H. (2001). Impaired processing of rapid stimulus sequences in dyslexia. *Trends Cogn. Sci.* 5 525–532 10.1016/S1364-6613(00)01801-5 11728910

[B44] Hari R. Renvall H. Tanskanen T. (2001). Left minineglect in dyslexic adults. *Brain* 124 1373–1380 10.1093/brain/124.7.1373 11408332

[B45] Hari R. Valta M. Uutela K. (1999). Prolonged attentional dwell time in dyslexic adults. *Neurosci. Lett.* 271 202–204 10.1016/S0304-3940(99)00547-9 10507704

[B46] Iles J. Walsh V. Richardson A. (2000). Visual search performance in dyslexia. *Dyslexia* 6 163–177 10.1002/1099-0909(200007/09)6:3<163::AID-DYS150>3.0.CO;2-U 10989565

[B47] Ito H. (2012). Illusory object motion in the centre of a radial pattern: the pursuit–pursuing illusion. *Iperception* 3 59–87 10.1068/i0430 23145267 PMC3485812

[B48] Jones M. W. Branigan H. P. Kelly M. L. (2008). Visual deficits in developmental dyslexia: relationships between non-linguistic visual tasks and their contribution to components of reading. *Dyslexia* 14 95–115 10.1002/dys.345 17874457

[B49] Kaplan E. Shapley R. (1982). X and Y cells in the lateral geniculate nucleus of macaque monkeys. *J. Physiol. (Lond.)* 330 125–143 7175738 10.1113/jphysiol.1982.sp014333 PMC1225290

[B50] Kaplan Keen A. G. Lovegrove W. J. (2000). Transient deficit hypothesis and dyslexia: examination of whole-parts relationship, retinal sensitivity, and spatial and temporal frequencies. *Vision Res. * 40 705–715 10.1016/S0042-6989(99)00208-4 10824271

[B51] Kelly D. (1966). Frequency doubling in visual responses. *JOSA* 56 1628–1632 10.1364/JOSA.56.001628

[B52] Kelly D. (1981). Nonlinear visual responses to flickering sinusoidal gratings. *JOSA* 71 1051–1055 10.1364/JOSA.71.001051 7277060

[B53] Kevan A. Pammer K. (2008). Visual deficits in pre-readers at familial risk for dyslexia. *Vision Res.* 48 2835–2839 10.1016/j.visres.2008.09.022 18929591

[B54] Kevan A. Pammer K. (2009). Predicting early reading skills from pre-reading measures of dorsal stream functioning. *Neuropsychologia* 47 3174–3181 10.1016/j.neuropsychologia.2009.07.016 19651148

[B55] Livingstone M. S. Hubel D. H. (1987). Psychophysical evidence for separate channels for the perception of form, color, movement, and depth. *J. Neurosci.* 7 3416–3468 3316524 10.1523/JNEUROSCI.07-11-03416.1987 PMC6569044

[B56] Livingstone M. S. Rosen G. D. Drislane F. W. Galaburda A. M. (1991). Physiological and anatomical evidence for a magnocellular defect in developmental dyslexia. *Proc. Natl. Acad. Sci. U.S.A.* 88 7943–7947 10.1073/pnas.88.18.7943 1896444 PMC52421

[B57] Lovegrove W. Martin F. Slaghuis W. (1986). A theoretical and experimental case for a visual deficit in specific reading disability. *Cogn. Neuropsychol.* 3 225–267 10.1080/02643298608252677

[B58] Luo H. Poeppel D. (2007). Phase patterns of neuronal responses reliably discriminate speech in human auditory cortex. *Neuron* 54 1001–1010 10.1016/j.neuron.2007.06.004 17582338 PMC2703451

[B59] Maddess T. Hemmi J. M. James A. C. (1992). Evidence for spatial aliasing effects in the Y-like cells of the magnocellular visual pathway. *Vision Res.* 38 1843–1859 10.1016/S0042-6989(97)00344-1 9797962

[B60] Marrocco R. T. McClurkin J. W. Young R A. (1982). Spatial summation and conduction latency classification of cells of the lateral geniculate nucleus of macaques. *J. Neurosci.* 2 1275–1291 7119875 10.1523/JNEUROSCI.02-09-01275.1982 PMC6564315

[B61] Martin F. Lovegrove W. (1987). Flicker contrast sensitivity in normal and specifically disabled readers. *Perception* 16 215–221 10.1068/p160215 3684483

[B62] Maunsell J. H. R. Newsome W. T. (1987). Visual processing in monkey extrastriate cortex. *Annu. Rev. Neurosci.* 10 363–401 10.1146/annurev.ne.10.030187.002051 3105414

[B63] Merigan W. H. Maunsell J. H. (1993). How parallel are the primate visual pathways? *Annu. Rev. Neurosci.* 16 369–402 10.1146/annurev.ne.16.030193.002101 8460898

[B64] Mishkin M. Ungerleider L. G. (1982). Contribution of striate inputs to the visuospatial functions of parieto-preoccipital cortex in monkeys. *Behav. Brain Res.* 6 57–77 10.1016/0166-4328(82)90081-X 7126325

[B65] Olulade O. A. Napoliello E. M. Eden G. F. (2013). Abnormal visual motion processing is not a cause of dyslexia. *Neuron* 79 1–11 10.1016/j.neuron.2013.05.002 23746630 PMC3713164

[B66] Pammer K. (2014). Temporal sampling in vision and the implication in dyslexia. *Front. Hum. Neurosci. * 7:1–18 10.3389/fnhum.2013.00933 PMC3925989 24596549

[B67] Pammer K. Wheatley C. (2001). Isolating the M (y)-cell response in dyslexia using the spatial frequency doubling illusion. *Vision Res.* 41 2139–2147 10.1016/S0042-6989(01)00092-X 11403797

[B68] Perry C. Ziegler J. C. Zorzi M. (2007). Nested incremental modeling in the development of computational theories: the CDP model of reading aloud. *Psychol. Rev.* 114 273–315 10.1037/0033-295X.114.2.273 17500628

[B69] Power A. J. Mead N. Barnes L. Goswami U. (2013). Neural entrainment to rhythmic speech in children with developmental dyslexia. *Front. Hum. Neurosci. * 7:777 10.3389/fnhum.2013.00777 PMC3842021 24376407

[B70] Roach N. W. Hogben J. H. (2007). Impaired filtering of behaviourally irrelevant visual information in dyslexia. *Brain* 130 771–785 10.1093/brain/awl353 17237361

[B71] Rochelle K. S. Witton C. Talcott J. B. (2009). Symptoms of hyperactivity and inattention can mediate deficits of postural stability in developmental dyslexia. *Exp. Brain Res.* 192 627–633 10.1007/s00221-008-1568-5 18830588

[B72] Ronconi L. Basso D. Gori S. Facoetti A. (2014). TMS on right frontal eye fields induces an inflexible focus of attention. *Cereb. Cortex* 24 396–402 10.1093/cercor/bhs319 23048022

[B73] Ronconi L. Gori S. Ruffino M. Franceschini S. Urbani B. Molteni M. (2012). Decreased coherent motion discrimination in autism spectrum disorder: the role of attentional zoom-out deficit. *PLoS ONE * 7:e49019 10.1371/journal.pone.0049019 PMC3490913 23139831

[B74] Ronconi L. Gori S. Ruffino M. Molteni M. Facoetti A. (2013a). Zoom-out attentional impairment in children with autism spectrum disorder. *Cortex* 49 1025–1033 10.1016/j.cortex.2012.03.005 22503282

[B75] Ronconi L. Gori S. Giora E. Ruffino M. Molteni M. Facoetti A. (2013b). Deeper attentional masking by lateral objects in children with autism. *Brain Cogn.* 82 213–218 10.1016/j.bandc.2013.04.006 23685759

[B76] Ruffino M. Gori S. Boccardi D. Molteni M. Facoetti A. (2014). Spatial and temporal attention are both sluggish in poor phonological decoders with developmental dyslexia. *Front. Hum. Neurosci. * 8:1–13 10.3389/fnhum.2014.00331 24904371 PMC4033052

[B77] Ruzzoli M. Gori S. Pavan A. Pirulli C. Marzi C. A. Miniussi C. (2011). The neural basis of the Enigma illusion: a transcranial magnetic stimulation study. *Neuropsychologia* 49 3648–3655 10.1016/j.neuropsychologia.2011.09.020 21952193

[B78] Ruffino M. Trussardi A. N. Gori S. Finzi A. Giovagnoli S. Menghini D. (2010). Attentional engagement deficits in dyslexic children. *Neuropsychologia* 8 3793–3801 10.1016/j.neuropsychologia.2010.09.002 20833191

[B79] Sartori G. Job R. Tressoldi P. E. (1995). *Batteria per la Valutazione Della Dislessia e Della Disortografia Evolutiva*. Firenze: Organizzazioni Speciali

[B80] Share D. L. (1995). Phonological recoding and self-teaching: sine qua non of reading acquisition. *Cognition* 55 151–218 10.1016/0010-0277(94)00645-2 7789090

[B81] Snowling M. (2000). *Dyslexia*. Oxford: Blackwell

[B82] Sperling A. J. Lu Z. Manis F. R. Seidenberg M. S. (2005). Deficits in perceptual noise exclusion in developmental dyslexia. *Nat. Neurosci.* 8 862–863 10.1038/nn1474 15924138

[B83] Sperling A. J. Lu Z. L. Manis F. R. Seidenberg M. S. (2006). Motion-perception deficits and reading impairment: it’s the noise, not the motion. *Psychol. Sci.* 17 1047–1053 10.1111/j.1467-9280.2006.01825.x 17201786

[B84] Sprenger-Charolles L. Siegel L. S. Béchennec D. Serniclaes W. (2003). Development of phonological and orthographic processing in reading aloud, in silent reading, and in spelling: a four-year longitudinal study. *J. Exp. Child Psychol.* 84 194–217 10.1016/S0022-0965(03)00024-9 12706384

[B85] Stein J. (2001). The magnocellular theory of developmental dyslexia. *Dyslexia* 7 12–36 10.1002/dys.186 11305228

[B86] Stein J. Walsh V. (1997). To see but not to read; the magnocellular theory of dyslexia. *Trends Neurosci.* 20 147–152 10.1016/S0166-2236(96)01005-3 9106353

[B87] Talcott J. B. Witton C. Hebb G. S. Stoodley C. J. Westwood E. A. France S. J. (2002). On the relationship between dynamic visual and auditory processing and literacy skills; results from a large primary-school study. *Dyslexia* 8 204–225 10.1002/dys.224 12455851

[B88] Talcott J. B. Witton C. McLean M. F. Hansen P. C. Rees A. Green G. G. (2000). Dynamic sensory sensitivity and children’s word decoding skills. *Proc. Natl. Acad. Sci. U.S.A.* 97 2952–2957 10.1073/pnas.040546597 10688885 PMC16036

[B89] Talcott J. B. Witton C. Stein J. F. (2013). Probing the neurocognitive trajectories of children’s reading skills. *Neuropsychologia* 51 472–481 10.1016/j.neuropsychologia.2012.11.016 23178212

[B90] Tallal P. (2004). Improving language and literacy is a matter of time. *Nat. Rev. Neurosci.* 5 721–728 10.1038/nrn1499 15322530

[B91] Valdois S. Gérard C. Vanault P. Dugas M. (1995). Peripheral developmental dyslexia: a visual attentional account? *Cogn. Neuropsychol.* 12 31–67 10.1080/02643299508251991

[B92] Vellutino F. R. Fletcher J. M. Snowling M. J. Scanlon D. M. (2004). Specific reading disability (dyslexia): what have we learned in the past four decades? *J. Child Psychol. Psychiatry* 45 2–40 10.1046/j.0021-9630.2003.00305.x 14959801

[B93] Vidyasagar T. R. (1999). A neuronal model of attentional spotlight: parietal guiding the temporal. *Brain Res. Rev.* 30 66–76 10.1016/S0165-0173(99)00005-3 10407126

[B94] Vidyasagar T. R. (2013). Reading in to neuronal oscillations in the visual system: implications for developmental dyslexia. *Front. Hum. Neurosci. * 7:811 10.3389/fnhum.2013.00811 PMC3841981 24348361

[B95] Vidyasagar T. R. Pammer K. (1999). Impaired visual search in dyslexia relates to the role of the magnocellular pathway in attention. *Neuroreport* 10 1283–1287 10.1097/00001756-199904260-00024 10363940

[B96] Vidyasagar T. R. Pammer K. (2010). Dyslexia: a deficit in visuo-spatial attention, not in phonological processing. *Trends Cogn. Sci.* 14 57–63 10.1016/j.tics.2009.12.003 20080053

[B97] Wechsler D. (1986). *WISC-R: Scala di Intelligenza Wechsler per Bambini Riveduta*. Firenze: Organizazioni Speciali

[B98] Williams M. Brannan J. Lartigue E. (1987). Visual search in good and poor readers. *Clin. Vis. Sci.* 1 367–371

[B99] Wimmer H. (1993). Characteristics of developmental dyslexia in a regular writing system. *Appl. Psycholinguist.* 14 1–33 10.1017/S0142716400010122

[B100] Wright B. A. Bowen R. W. Zecker S. G. (2000). Nonlinguistic perceptual deficits associated with reading and language disorders. *Curr. Opin. Neurobiol.* 10 482–486 10.1016/S0959-4388(00)00119-7 10981617

[B101] Yazdanbakhsh A. Gori S. (2008). A new psychophysical estimation of the receptive field size. *Neurosci. Lett.* 438 246–251 10.1016/j.neulet.2008.04.040 18467028 PMC2483954

[B102] Yazdanbakhsh A. Gori S. (2011). Mathematical analysis of the accordion grating illusion: a differential geometry approach to introduce the 3D aperture problem. *Neural Netw.* 24 1093–1101 10.1016/j.neunet.2011.06.016 21782387

[B103] Ziegler J. C. Perry C. Ma-Wyatt A. Ladner D Schulte-Körne G. (2003). Developmental dyslexia in different languages: language-specific or universal? *J. Exp. Child Psychol.* 86 169–193 10.1016/S0022-0965(03)00139-5 14559203

[B104] Zorzi M. Barbiero C. Facoetti A. Lonciari I. Carrozzi M. Montico M. (2012). Extra-large letter spacing improves reading in dyslexia. *Proc. Natl. Acad. Sci. U.S.A.* 109 11455–11459 10.1073/pnas.1205566109 22665803 PMC3396504

